# Early Diagnosis of Primary Right Atrial Angiosarcoma From Intraoperative Transesophageal Echocardiographic Findings of Right Atrial Pseudoaneurysm and Cardiac Rupture

**DOI:** 10.7759/cureus.73521

**Published:** 2024-11-12

**Authors:** Masaru Shimizu, Taiken Banno, Misayo Nishikawa

**Affiliations:** 1 Anesthesiology, Uji Tokushukai Medical Center, Uji, JPN

**Keywords:** cardiac angiosarcoma, cardiac malignancy, pericardial effusion, pericardiotomy, pseudoaneurysm, transesophageal echocardiography

## Abstract

Primary cardiac angiosarcoma is a rare and aggressive malignant tumor with a poor clinical outcome. Its nonspecific symptoms often complicate early diagnosis. Here, we present the case of a 39-year-old man who initially presented with pericardial effusion and cardiac tamponade. Despite multiple transthoracic echocardiography (TTE) and computed tomography (CT) scans from admission to surgery, no cardiac tumor was detected. After admission, pericardial drainage was performed following the parasternal approach. On day 5 after admission, TTE and chest CT revealed increased pericardial effusion compared to post-drainage on admission. On day 6 of admission, the patient underwent a left pericardial window creation under general anesthesia. We did not suspect malignancy until an intraoperative transesophageal echocardiography (TEE) was performed, which revealed a right atrial pseudoaneurysm despite subsequent negative imaging results. On day 7 of admission, the patient was treated with surgical resection of the pseudoaneurysm and repair of the right atrial wall, followed by a histopathologic diagnosis of cardiac angiosarcoma. The patient recovered without major complications, was discharged from the hospital, and is doing well. Early surgical intervention, as evidenced in this case, can substantially improve patient outcome. This report provides valuable insights into the management of cardiovascular angiosarcoma. We emphasize the importance of a multifaceted, comprehensive diagnostic evaluation that includes the use of advanced imaging modalities, such as TEE, to make a timely and accurate diagnosis, even in the absence of obvious tumor findings.

## Introduction

Approximately 30% of primary cardiac tumors, including angiosarcoma, lymphoma, fibrosarcoma, and myosarcoma, are malignant [1]. Among these, angiosarcoma is the most common primary cardiac malignancy in adults [2]. Its aggressive nature and rapid progression contribute to a poor prognosis, and the clinical symptoms often complicate timely diagnosis. Advances in imaging studies in recent years have improved the early detection of cardiac tumors, aiding in precise perioperative management [3].

This case presented a rare manifestation of a right atrial angiosarcoma where no clear tumor mass was identifiable on imaging. The strength of this study lies in the detailed documentation of clinical, surgical, and pathologic findings, providing valuable insight into the diagnosis and management of such a rare case. Our findings highlight the necessity of a comprehensive perioperative diagnostic assessment, even in the absence of a discernible mass on imaging.

## Case presentation

A 39-year-old man presented with symptoms of fever, fatigue, and epicardial pain for four days and subsequently developed persistent dyspnea. Transthoracic echocardiography (TTE) revealed a significant pericardial effusion with good left ventricular contractility and no regurgitation of the aortic, mitral, or tricuspid valves. He was subsequently transferred to our hospital for treatment. The patient’s medical history included a herniated lumbar disc and a family history that included a grandfather who suffered a myocardial infarction. On admission, his physical examination showed a pulse rate of 103 beats/minute, blood pressure of 95/69 mmHg, respiratory rate of 20 breaths/minute, and oxygen saturation (SpO2) of 100% on room air. No jugular vein distention or edema of the lower extremities was observed. Post-hospitalization blood tests revealed liver and renal dysfunction and elevated inflammatory response (Table 1).

**Table 1:** Blood test results upon hospitalization. PT: prothrombin time; INR: international normalized ratio; APTT: activated partial thromboplastin time

Test	Results	Normal Range
White Blood Cells (× 10^2^/µL)	232	33 - 86
Hemoglobin (g/dL)	13.2	13.7 - 16.8
Hematocrit (%)	40.8	40.7 - 50.1
Platelet (× 10^4^/μL)	19.9	15.8 - 34.8
PT-INR	1.35	0.9 - 1.1
APTT (seconds)	30.9	24 - 34
Fibrinogen (mg/dL)	268	200 - 400
C-Reactive Protein (mg/dL)	0.71	0 - 0.14
Creatine Phosphokinase (U/L)	123	59 - 248
Creatine Kinase Muscle-Brain (U/L)	25	4 - 16
Sodium (mmol/L)	140	138 - 145
Potassium (mmol/L)	3.9	3.6 - 4.8
Chloride (mmol/L)	109	101 - 108
Urea Nitrogen (mg/dL)	11.3	8. 0 - 20. 0
Creatinine (mg/dL)	1.49	0. 65 - 1. 07
Aspartate Aminotransferase (U/L)	427	13 - 30
Alanine Aminotransferase (U/L)	379	10 - 42
Lactate Dehydrogenase (U/L)	812	124 - 222
Total Bilirubin (mg/dL)	0.63	0.4 - 1.5
Total Protein (g/dL)	5.4	6.6 - 8.1
Albumin (g/dL)	3.3	4.1 - 5.1

**Figure 1:**
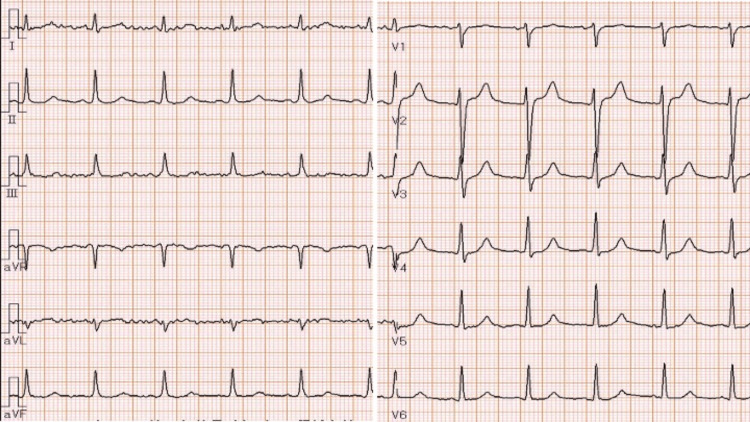
Electrocardiogram upon admission showing sinus rhythm with normal PR, QTc intervals, no ST-T segment changes, and heart rate of 98 beats per minute.

**Figure 2:**
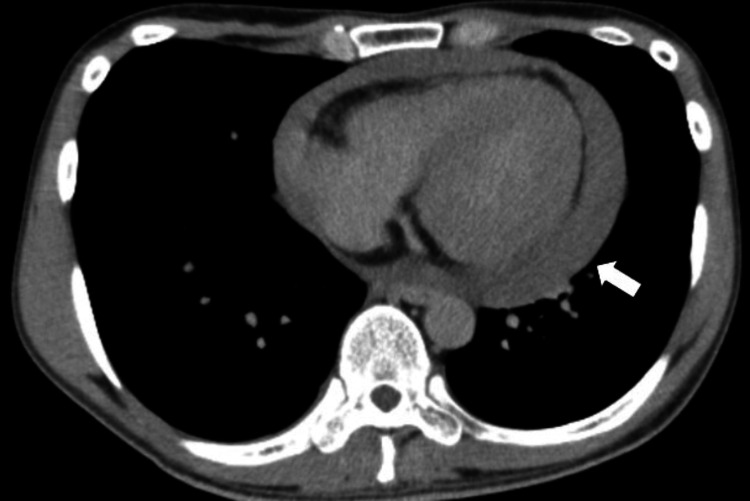
Chest CT (axial view) upon admission revealing significant pericardial effusion, due to increased pericardial fluid accumulation around the heart. The effusion is notable in the posterior region of the pericardium (arrow).

Electrocardiography (ECG) showed sinus rhythm with no pathologic ST-T segment changes (Figure 1), and chest CT showed pericardial effusion without cardiac masses, pulmonary nodules, or metastases (Figure 2).

**Figure 3:**
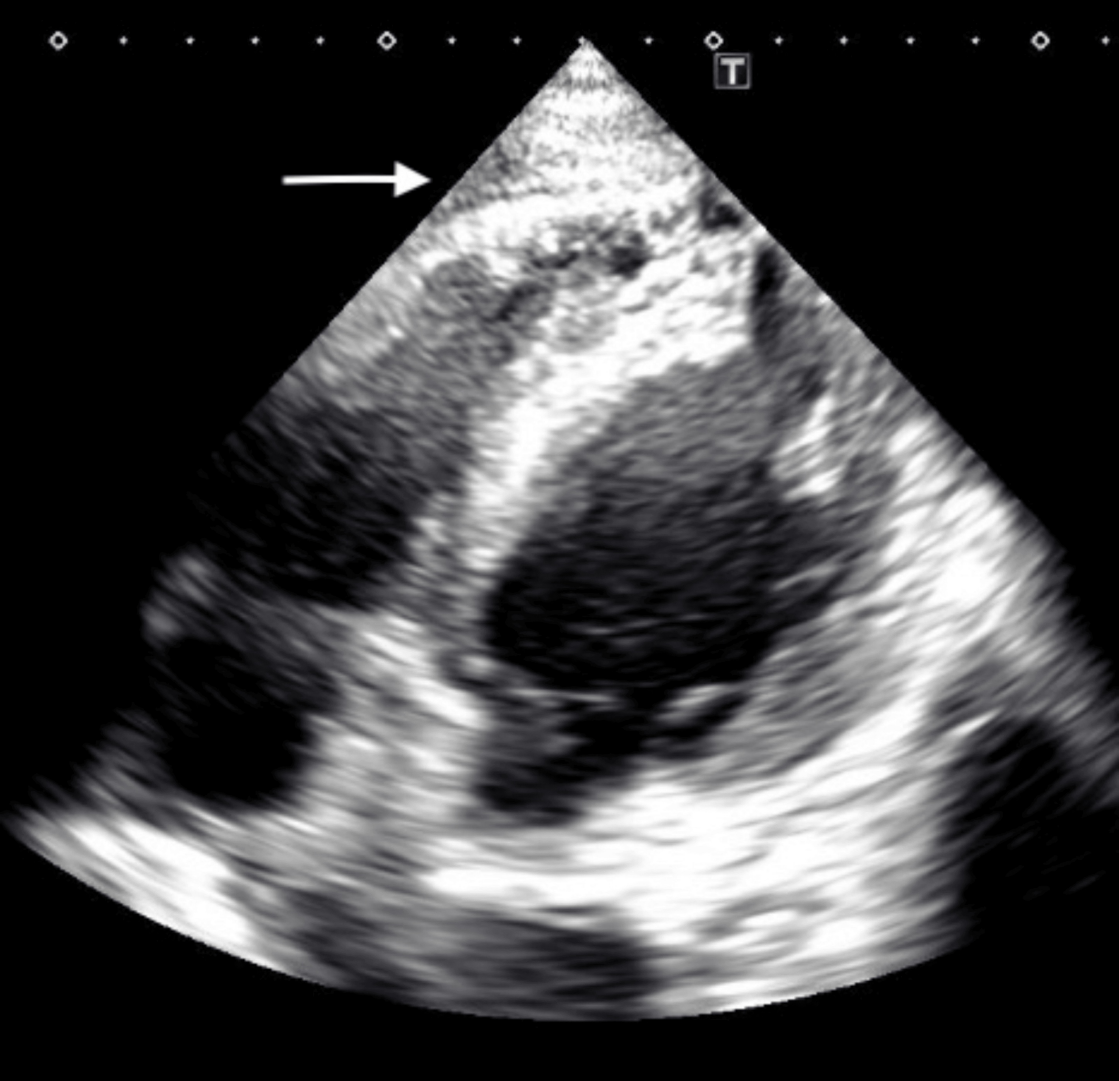
Transthoracic echocardiography after pericardiocentesis revealing a small amount of pericardial fluid (arrow).

Pericardial drainage with the parasternal approach was performed to drain 1,300 ml of bloody pericardial fluid, and subsequent continuous drainage was performed. The patient’s pulse rate decreased to 74 beats/minute, and blood pressure increased to 114/77 mmHg. TTE showed normal left ventricular function, with no cardiac enlargement, abnormal septal motion, or masses in the pericardial cavity (Figure 3, Video 1).

**Video 1:** Transthoracic echocardiography after pericardiocentesis showing no evidence of right atrial enlargement or abnormal left ventricular wall motion.

**Figure 4:**
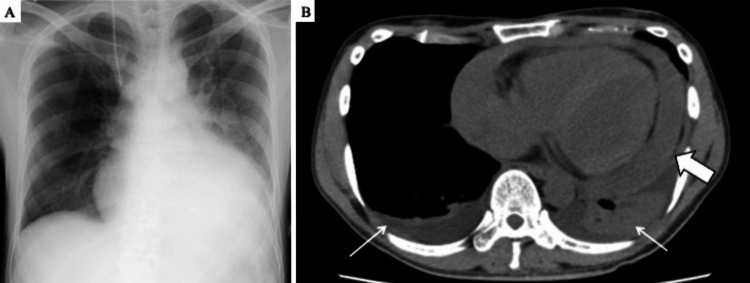
Imaging after five days of admission. (A) Chest radiograph showing an enlarged cardiac shadow with a cardiothoracic ratio of 66%. (B) Chest CT (axial view) showing pericardial effusion (arrow) and bilateral pleural effusions (narrow arrow).

**Figure 5:**
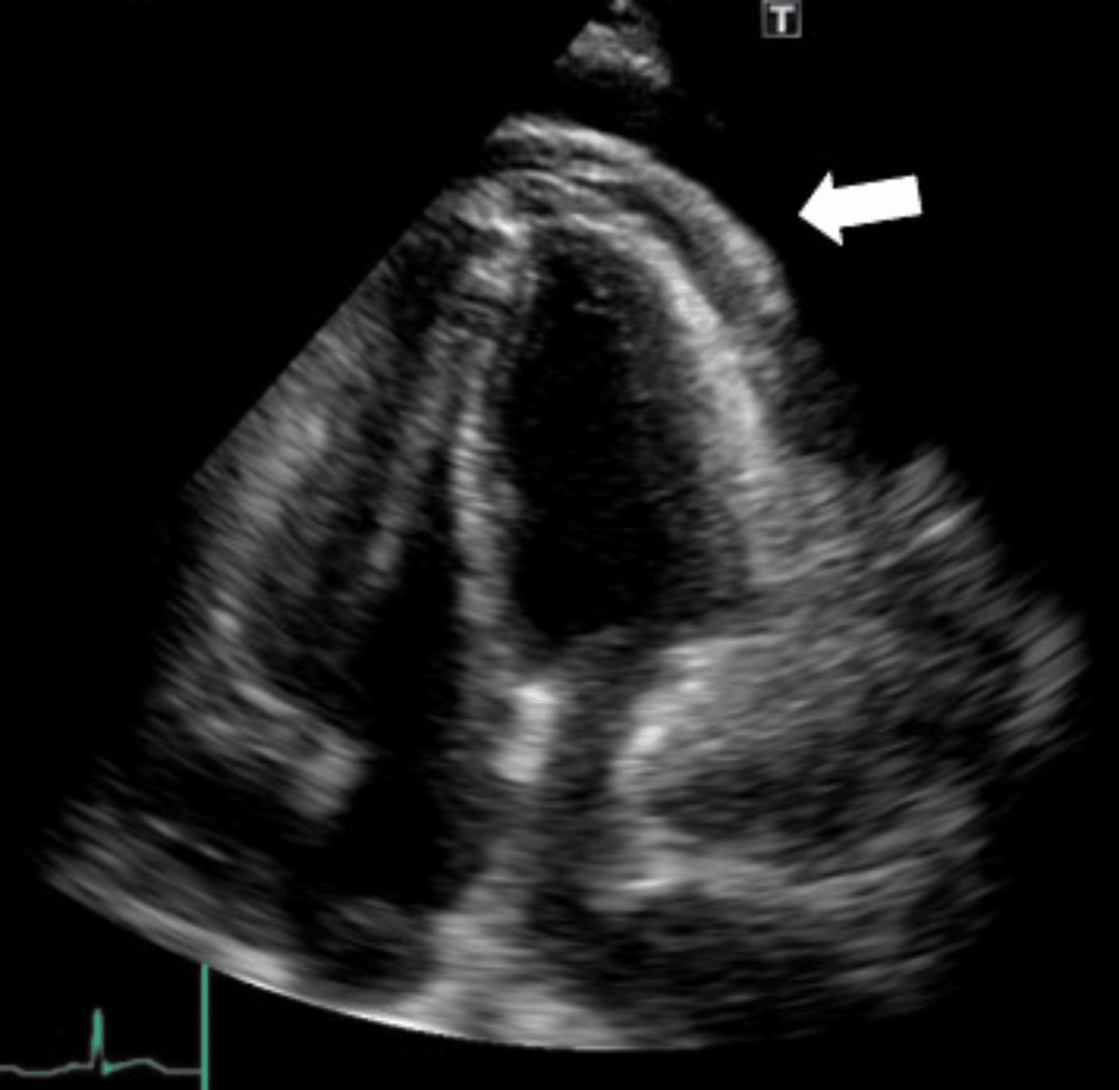
Transthoracic echocardiography after five days of admission showing increased pericardial effusion (arrow).

Cytological analysis of the pericardial fluid was negative for malignancy and infection, leading to a working diagnosis of infectious pericarditis based on chest pain, dyspnea, and elevated inflammatory marker. The patient was started on meropenem (15.9 mg/kg/day) and vancomycin (23.8 mg/kg/day). Nasal high-flow oxygen therapy (NHF) was administered on day 1 of admission because of poor oxygenation but this treatment was discontinued the following day. On day 3, repeated TTEs revealed the presence of a circumferential pericardial effusion; however, as the patient was not symptomatic, he was observed. On day 5, chest radiography and CT scan revealed cardiac enlargement (Figure 4), and TTE revealed increased pericardial effusion compared to the previous examination (Figure 5).

**Figure 6:**
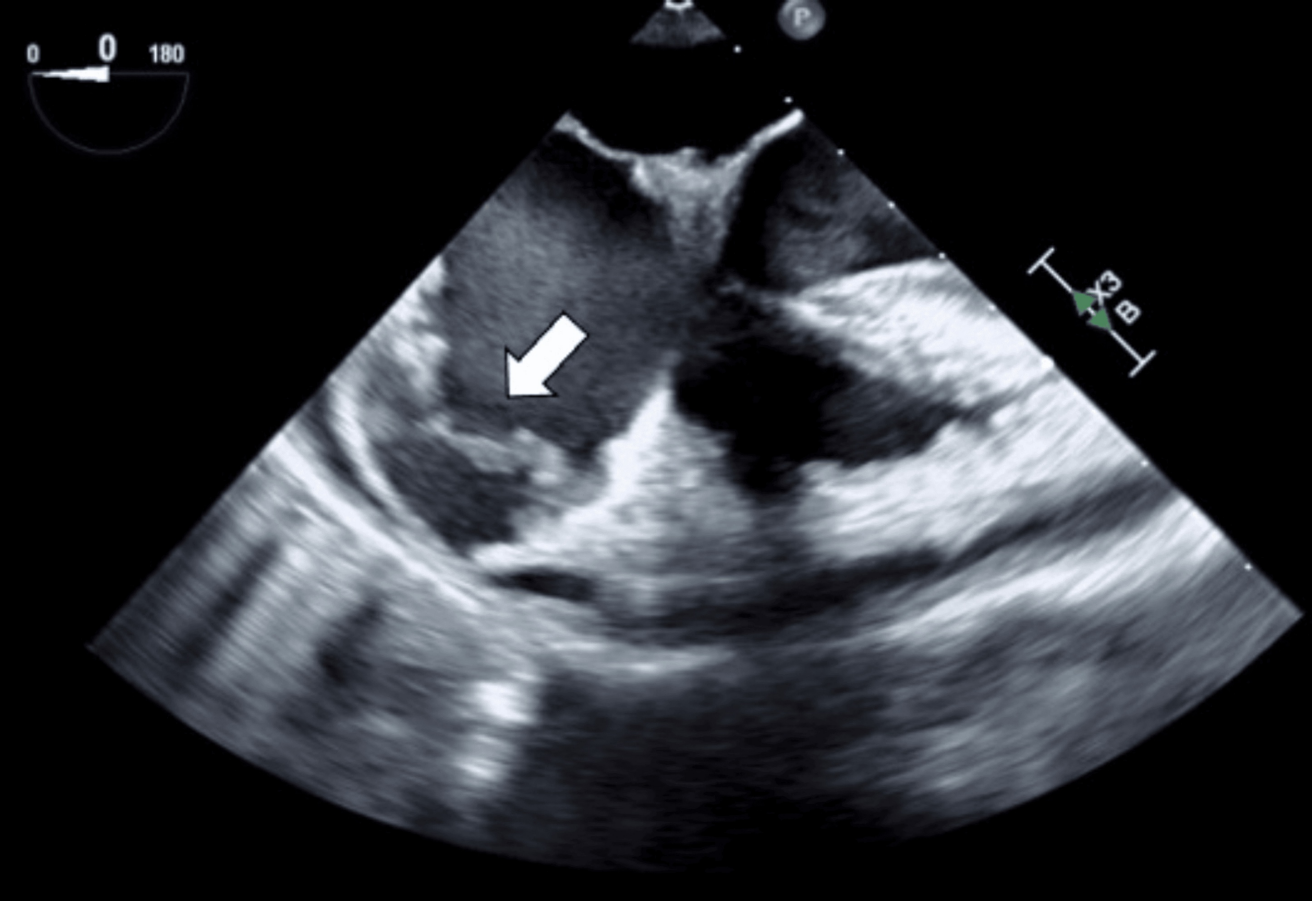
Intraoperative TEE in mid-esophageal four-chamber view during pericardial window surgery revealing a right atrial pseudoaneurysm (arrow) without an obvious tumor In this image, the TEE probe was rotated clockwise from the standard mid-esophageal four-chamber view, primarily highlighting the structures of the right atrium and right ventricle. TEE: transesophageal echocardiography

Thus, the previous pericardial drainage catheter was replaced with a new one; however, effective drainage was not possible. As the pericardial effusion did not improve, which was evident on the chest CT scan, he was scheduled for the creation of a left pericardial window under general anesthesia on day 6 of admission. The induction of anesthesia was performed with attention to hypervasodilation and circulatory dynamics. Rapid induction was performed using 1 mg/kg propofol, 3 μg/kg fentanyl, 0.3 μg/kg/minute remifentanil, and 0.8 mg/kg rocuronium. The patient was intubated, and a transesophageal echocardiography (TEE) was inserted. No significant circulatory changes were observed. TEE showed a right atrial pseudoaneurysm but no tumor (Figure 6).

The surgery was approached through the left sixth intercostal pleural opening. Pericardiotomy was undertaken, and 1,300 ml of pericardial blood was drained; then, a drain was placed in the left thoracic cavity. Anesthesia lasted for 110 minutes, with a total surgery time of 53 minutes. After surgery, the patient was transferred to the intensive care unit under sedation.

**Figure 7:**
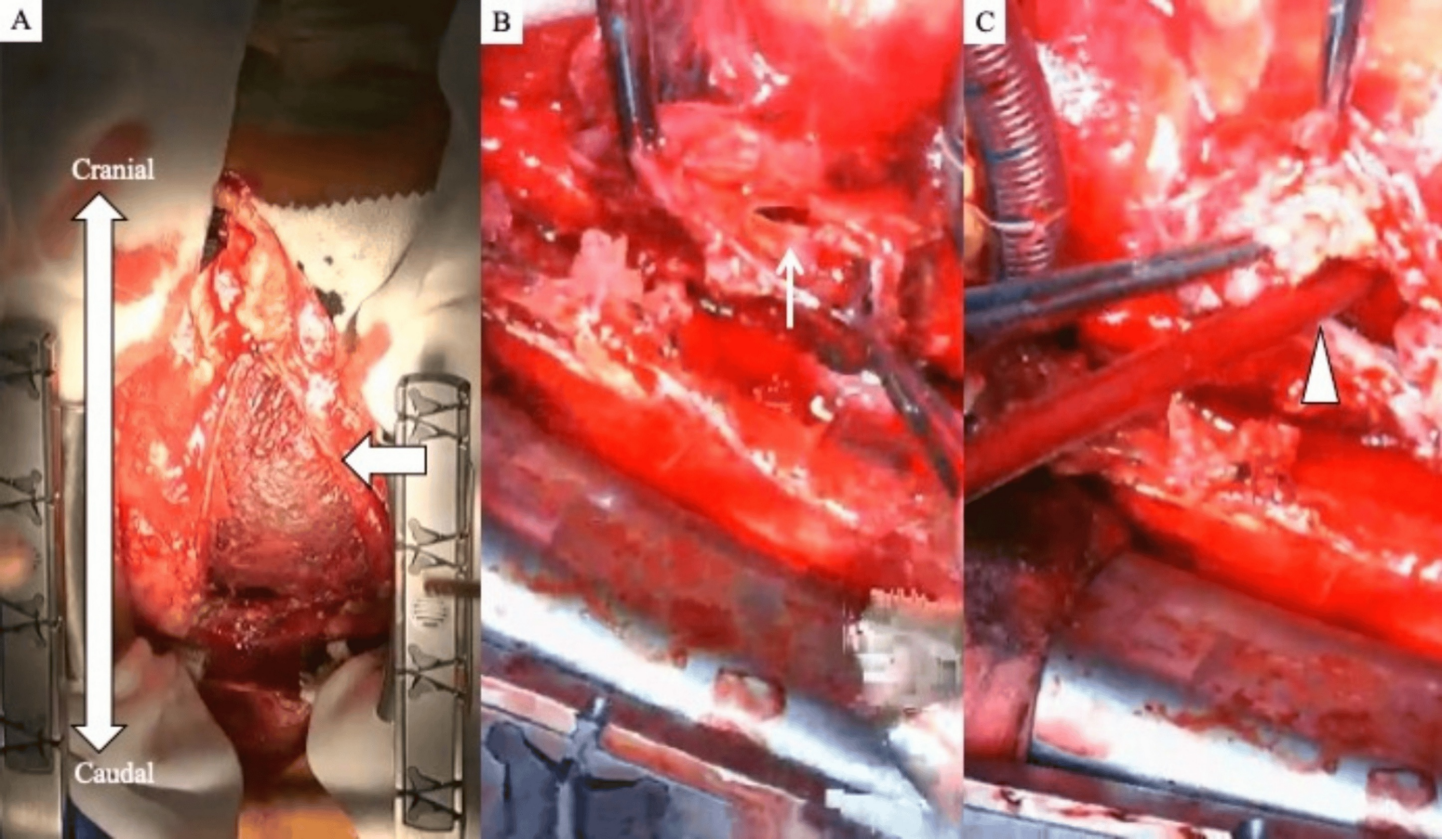
Intraoperative findings. (A) A hematoma and fibrin clot are seen on the anterior surface of the right atrium (arrow). (B) The right atrial wall is ruptured (narrow arrow). (C) Degeneration and thickening of part of the right atrial wall, resulting in grossly abnormal tissue (arrowhead).

The patient was then scheduled for resection of the right atrial pseudoaneurysm and repair of the right atrial wall the following day. The procedure was performed via a median sternotomy under general anesthesia. The induction of anesthesia did not result in any hemodynamic alterations or reduction in blood pressure. At our hospital, a pulmonary artery catheter is typically inserted during cardiac surgery, but we judged that insertion would be difficult in this case because of right atrial disease. Cardiopulmonary bypass was initiated following cannulation of the ascending aorta, superior vena cava, and inferior vena cava. Intraoperative findings showed a rupture of the right atrial wall with pseudoaneurysm formation after the removal of a hematoma and fibrin clot in the anterior right atrium (Figures 7A, 7B). The right atrial wall around the rupture site was degenerated and thickened (Figure 7C).

The abnormal tissue was excised up to grossly normal areas, and the defect was repaired with a bovine pericardial patch (Model 4700; Edwards Lifesciences Corporation, Irvine, California, United States) [4]. Anesthesia time was 202 minutes, total surgery time was 142 minutes, and cardiopulmonary bypass time was 49 minutes. Blood loss was 640 mL during the surgery; eight units of red blood cells and six units of fresh frozen plasma were transfused, as the hemoglobin level decreased to 7.5 g/dL. After surgery, he was again transferred to intensive care. The right atrial wall tissues resected during the surgery were submitted for histopathological examination. Since a part of the right atrium was abnormal, we considered the possibility of a tumor and performed head, chest, and abdomen CT scans postoperatively. No metastasis to other organs was observed. The patient progressed and recovered without major complications and was discharged on day 23 of admission.

**Figure 8:**
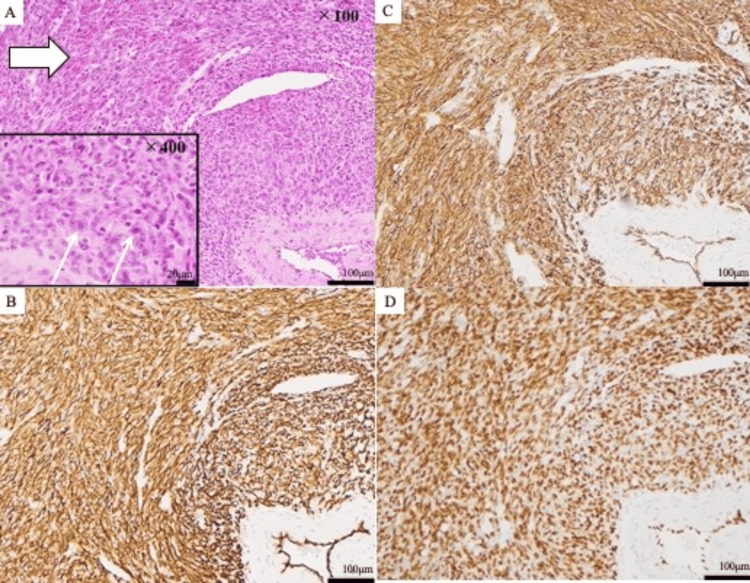
Histopathologic findings. Microscopic findings show an extensive, poorly formed tumor, with anastomosing vascular channels containing prominent blood (arrow), mild nuclear atypia, frequent formation of slit-like vascular spaces, and pleomorphic atypical cells proliferating in fullness (narrow arrow); hematoxylin & eosin stain, original magnification 100× and  400× (A). The immunohistochemistry findings were positive for CD31 (B), CD34 (C), and ERG (D).

Histopathological examination revealed the presence of oval and spindle-shaped cells in the abnormal tissue and diffuse proliferation of atypical cells with vesicular nuclei and small nucleoli in a bundle-like arrangement, as seen in scattered nuclear fission images (Figure 8A). Therefore, a high-grade malignant mesenchymal tumor was suspected. Immunohistochemistry was positive for CD31, CD34, and ERG, with a Ki67 proliferation index of approximately 60% (Figures 8B-8D), confirming the diagnosis of angiosarcoma.

Negative results for AE1/AE3, desmin, and S-100 ruled out epithelial tumors, myxoid tumors, malignant melanoma, and mesothelioma. Ultimately, we diagnosed angiosarcoma in the right atrial tissue. Approximately five months after surgery, the patient demonstrated positive outcomes in terms of recovery. At the patient's choice, no additional chemotherapy or immunotherapy was administered during this period. The timeline of events is shown in Table 2.

**Table 2:** Clinical course. Major events from symptom onset to discharge. NHF: nasal high-flow oxygen; TEE: transesophageal echocardiography; ICU: intensive care unit

Day	Events
Four days before admission	Onset of fever, fatigue, and epigastric pain.
Day 0 (Admission)	Due to cardiac tamponade, 1300 mL of hemorrhagic pericardial fluid is drained via pericardiocentesis. Pericarditis was suspected, and the antibiotic treatment was started.
Day 1	Symptoms improved, but oxygenation worsened, and NHF therapy was started.
Day 4	Oxygenation improved, and oxygen therapy was discontinued.
Day 5	Chest radiograph showed cardiomegaly. Pericardial drain was re-inserted.
Day 6	Due to poor pericardial drainage, a pericardial window procedure was performed. Intraoperative TEE revealed a right atrial pseudoaneurysm and rupture.
Day 7	Right atrial repair was performed. Right atrial tissue was submitted for histopathological examination.
Day 8	Extubation was performed in the ICU.
Day 23	Postoperative course was uneventful. The patient was discharged from the hospital.
Day 27	Histopathological diagnosis confirmed cardiac angiosarcoma.

## Discussion

Primary cardiac angiosarcoma is a rare, infiltrative endothelial cell tumor that predominantly affects the myocardial wall, with an autopsy incidence of less than 0.03% [5]. It is one and a half times more prevalent in male than in female patients, typically affecting individuals aged 20-50 years [6]. As a high-grade tumor, approximately 75% of cases originate in the right atrium, often invading the pericardium, tricuspid valve, right ventricle, and right coronary artery, with metastasis to other organs, particularly the lungs, in up to 90% of cases [7].

While the etiology of angiosarcoma remains largely unknown, several potential risk factors such as prior radiation therapy and excessive ultraviolet radiation from prolonged sun exposure have been identified [8]. Patients with cardiac angiosarcoma are typically asymptomatic or show non-specific symptoms, which include dyspnea, chest pain, palpitations, and weight loss. Symptoms depend on the tumor size, anatomic location, fragility, embolic tendencies, growth rate, invasion, and metastasis [9].

The most common complication is pericardial effusion, observed in more than half of cases. Other complications include vena cava obstruction, arrhythmias, and chronic heart failure [1]. Pericardial fluid is mostly bloody but often lacks malignant cells [10], limiting the utility of pericardial fluid cytology in ruling out malignancy. Cytological examination results of the patient's pericardial fluid on multiple occasions were negative for malignant cells.

The causes of pseudoaneurysms and cardiac rupture are varied, including malignancy-induced myocardial necrosis, iatrogenic injury, trauma, or myocardial infarction [11]. Cardiac rupture in angiosarcoma is extremely rare, with few reports of survival [12]. The prognosis remains poor due to the rarity and non-specificity of disease symptoms, which often delay the diagnosis. Early detection is crucial since a delay can worsen outcomes.

Diagnostic approaches for cardiac angiosarcoma include echocardiography, CT, MRI, and histopathology [13]. TTE is a convenient, noninvasive, cost-effective, initial test with a sensitivity of 82% for cardiac tumor visualization [14]. TEE provides enhanced image resolution offering more detailed information about tumor location, characteristics, extent, site of attachment, wall invasion, and extension into the cardiac cavity [15]. Additional diagnostic modalities include CT, cardiac MRI (CMR), and positron emission tomography (PET)-CT. Multidetector CT (MDCT) provides information on a wide range of tumors, including myocardial, pericardial, and mediastinal invasion of cardiac tumors, as well as extension into the great vessels and pulmonary metastases. On contrast-enhanced CT, cardiac angiosarcomas are seen as heterogeneous masses with infiltration of the cardiac muscle and pericardium, often with pericardial effusion and thickening [16]. However, the contrast enhancement pattern of the tumor does not predict the grade of the angiosarcoma. CMR offers high temporal and spatial resolution and can provide excellent non-invasive tissue characterization. It is useful for assessing cardiac function and tumor and morphological characteristics and shows high specificity for differentiating between tumors and blood clots, making it the gold standard technique in evaluating cardiac tumors [17]. PET-CT is valuable for assessing tumor grade, recurrence, and metastasis, achieving 100% sensitivity [18].

In this case, preoperative TTE and CT failed to confirm the presence of a tumor or identify the cause of the pericardial effusion. However, intraoperative TEE allowed a definitive diagnosis and treatment planning. Most cases reveal tumors on imaging, facilitating diagnosis; however, this case underscores the need for comprehensive imaging when faced with unexplained cardiac abnormalities.

The definitive diagnosis of cardiac angiosarcoma requires a tissue sample for histopathological examination and immunohistochemistry. According to the 2020 World Health Organization soft tissue tumor classification, essential diagnostic criteria include CD31 and ERG expression in immunohistochemistry [8]. The ERG oncoprotein, an ETS (Erythroblast Transformation Specific) family transcription factor, is a highly specific and sensitive marker for angiosarcoma [19]. Other histopathologic features specified in the mandatory criteria are angiogenesis, multilayering of endothelial cells, nuclear anaplasia, increased mitosis, and necrosis.

The mean survival for cardiac angiosarcomas is 3.8 ± 2.5 months without surgical resection. When combined with surgery and preoperative chemotherapy, the median overall survival increases to 27 months, with a maximum survival of 9.5 years [7]. No treatment guidelines for primary cardiac angiosarcoma exist; treatment options include surgery, chemotherapy, and radiation therapy. In the absence of metastases, surgical resection remains the preferred first-line treatment [16]. Aggressive complete resection results in a good one-year survival rate of 80% [20]. Chemotherapy and radiation therapy are options for patients in whom complete surgical resection is not possible [7].

Physicians must remain vigilant for atypical situations, as early and accurate diagnosis of cardiac angiosarcoma can significantly impact treatment outcomes. This case demonstrates the role of early surgical intervention, which allowed for tumor removal despite its minimal visibility, potentially improving the patient's prognosis. The lack of long-term follow-up data limits our ability to assess the recurrence and long-term outcomes associated with these cases, underscoring the need for larger studies with extended follow-up to validate these findings.

## Conclusions

We present a rare and challenging case of cardiac angiosarcoma with pseudoaneurysm formation and cardiac rupture, identified and managed despite the absence of a visible tumor mass on imaging. This case highlights the importance of considering angiosarcoma in the differential diagnosis, even in the absence of an obvious mass on imaging. An unexplained cardiac abnormality should prompt more comprehensive imaging and the consideration of a possible malignancy. This case highlights the value of a thorough and multifaceted diagnostic approach in such rare cases. Finally, this report adds value to the existing literature in the field and is expected to help clinicians recognize and manage similar cases in the future.
